# The Effects of Mental Fatigue on Anaerobic Power and Power Endurance Performance

**DOI:** 10.3390/sports12070192

**Published:** 2024-07-16

**Authors:** Matthew P. Gonzalez, Denver M. Y. Brown, Isabella M. Swafford, Bryce Summerville, Morteza Seidi, Marzieh Hajiaghamemar, Sandor Dorgo

**Affiliations:** 1 Department of Kinesiology, The University of Texas at San Antonio, San Antonio, TX 78249, USA; sandor.dorgo@utsa.edu; 2 Department of Psychology, The University of Texas at San Antonio, San Antonio, TX 78249, USA; denver.brown@utsa.edu (D.M.Y.B.); isabella.swafford@my.utsa.edu (I.M.S.); bryce.summerville@my.utsa.edu (B.S.); 3 Department of Mechanical Engineering, The University of Texas at San Antonio, San Antonio, TX 78249, USA; morteza.seidi@utsa.edu; 4 Department of Biomedical Engineering, The University of Texas at San Antonio, San Antonio, TX 78249, USA; marzieh.memar@utsa.edu

**Keywords:** cognitive control, strength training, ego depletion, physical performance

## Abstract

Mental fatigue has been studied extensively in relation to its impact on aerobic-, strength-, and motor-based tasks, but anaerobic power-based tasks have received limited attention. Interdisciplinary research investigating the underlying mechanisms by which mental fatigue influences physical performance has been called for. In two studies, the effects of mental fatigue on maximal power jump and endurance jump performance as well as kinetics and kinematics during jump performance were examined. Samples of collegiate volleyball players (Study 1; N = 14) and recreationally active students (Study 2; N = 27) completed two 30 min experimental manipulations (high vs. low cognitive control exertion) before performing three maximal power squat jumps followed by 15 repeated countermovement jumps, with measurements of kinetics and kinematics. For Study 1, the maximal power performance was significantly lower under a mentally fatigued condition, but no differences were observed for repeated jump performance, which may have been attributable to alterations in jump mechanics. For Study 2, no between-condition differences were observed for the maximal power performance, although repeated jump performance was significantly lower under a mentally fatigued condition. Collectively, these findings suggest that the impacts of mental fatigue on power-based performance tasks may depend on the task demands as well as the training status of the individual.

## 1. Introduction

Psychological factors play an important role in achieving peak physical performance. Within this area of study, there is an emerging interest in the carryover effects of mental fatigue on physical performance [1]. Such work acknowledges that throughout the course of our day, we consistently encounter tasks involving high cognitive demands—whether it be a student studying for an exam, an employee designing a complex spreadsheet, or even a spouse trying to navigate a challenging conversation with their partner. Experiences that require cognitive resources can leave us feeling mentally drained, a state referred to as mental fatigue [2]. For athletes, mental fatigue has the potential to disrupt their performance in the training room, at practice, and during competitions. Therefore, understanding the magnitude of these effects as well as how and why this happens is imperative for optimizing human performance.

Initial research investigating the downstream effects of mental fatigue on subsequent physical performance involved highly controlled tasks that lacked external validity (e.g., isometric handgrip test) [3], but this research field has since evolved to examine physical tasks more commonly used in fitness (e.g., resistance training, aerobic exercise) and sports (e.g., soccer, shooting, cycling) [4]. As this body of literature has rapidly expanded, systematic reviews and meta-analyses have been conducted to quantify the direction and magnitude of the relationship between mental fatigue and physical performance. Meta-analytic evidence from these studies has shown that mental fatigue has small to medium-sized negative effects (*d* = −0.38 to −0.55) on physical performance depending on the comprehensiveness of the literature included [4,5,6]. Given the breadth of physical modalities that have been studied to date, it is important to consider that these effects may differ depending on the requirements of the task as well as the training status of the individual, as Jaydari Fard and colleagues reported that mental fatigue impaired non-athletes’ reaction time to a greater extent than that of athletes [7]. Similarly, Martin and colleagues suggested that professional cyclists have greater resistance to the impact of mental fatigue than recreational cyclists [8].

Pageaux and Lepers [9] were the first to synthesize the body of literature investigating the relationship between mental fatigue and physical performance. The findings from their systematic review revealed that mental fatigue may have differential effects depending on the type of physical task performed. Specifically, they found that negative carryover effects of mental fatigue were more consistently observed and were of greater magnitude during tasks that require prolonged submaximal effort regulation (e.g., self-paced exercise, time-to-exhaustion trials) compared to those involving brief maximal effort (e.g., 100 m sprint, long jump). These findings have since been supported by meta-analytic evidence [4], in that a null effect was observed for power-based tasks (*g* = 0.10), whereas significant negative effects were observed for aerobic- (*g* = −0.26), strength- (*g* = −0.51) and motor-based (*g* = −0.57) tasks. To date, however, the overwhelming majority of studies have investigated aerobic-, strength- and motor-based tasks—only two of the 91 effect sizes included in Brown’s [4] meta-analysis were from power-based anaerobic tasks. Thus, concluding that mental fatigue does not affect power-based tasks is premature. Without further studies, athletes and their coaches could be misled into believing power and power endurance performance is not impacted by fatigue when it may be.

While it is important to quantify the impact of mental fatigue on various indices of physical performance, it is also vital that we understand the mechanisms driving these effects so that we can develop appropriate interventions to circumvent performance impairments. From a psychological standpoint, research supports the idea that the perception of effort is the primary determinant of physical performance [10]. On the other hand, studies using metabolic cart systems and electromyography to assess physiological responses have, for the most part, failed to identify factors that govern performance in a mentally fatigued state [1]. While psychology and physiology represent two key pillars of human performance, a more comprehensive approach needs to be taken to advance our understanding.

The field of biomechanics has made substantial contributions to our understanding of human performance through quantifying the implications of how we move. To date, however, motion capture techniques have rarely been applied to examine whether mental fatigue causes changes in movement kinematics during the performance of physical tasks. In the one study utilizing these techniques, Kong and colleagues found differences in ankle stiffness, ankle inversion, loading rate, and knee valgus in injured limbs following unexpected side step cutting in mentally fatigued conditions, but did not find these differences when performing expected side step cutting tasks [11]. It is reasonable to postulate that when mentally fatigued, athletes may move in less efficient ways that compromise their performance, potentially placing them at increased risk of injury at the same time. Researchers in the field of exercise psychology have acknowledged that interdisciplinary collaborations with biomechanists are an important next step in our efforts to gain novel insights into the mechanistic pathways by which mental fatigue impairs physical performance [12].

The body of literature investigating the impact of physical fatigue on movement kinematics lends insights into what could be expected with regard to the potential downstream effects of mental fatigue on anaerobic performance. Specifically, alterations in the kinematics of the hip, knees, and ankles have been observed prior to the take-off phase of a vertical jump in a physically fatigued state [13]. Decreases in knee flexion and ankle dorsiflexion were also observed during a sequence of repeated jumps, which may have contributed to the reductions in peak force values that were found [13]. Similarly, Cooper and colleagues found that physical fatigue imparted negative effects on jump height and peak force during squat jumps and countermovement jumps [14]. While physical fatigue has received considerable attention regarding its effects on movement kinematics during anaerobic task performance, it is currently unknown as to whether similar findings would be observed under mentally fatigued conditions. The few studies that have examined the effects of mental fatigue on vertical jump performance have found no differences in jump performance resulting from mental fatigue, but have measured vertical jumps through cell phone applications [15,16], contact mats [17,18], and linear encoders [19], thus not allowing for an examination of kinematic changes during this movement, and as such it is unclear if there are alterations in jump mechanics resulting in the lack of differences during mentally fatigued conditions. Moreover, it has been suggested that mental fatigue affects more highly demanding cognitive tasks [16], but more demanding physical tasks, such as a repeated jump tasks, have yet to be examined.

Thus, the purpose of this study was to examine the effects of mental fatigue on vertical jump and repeated jump tasks, as well as their kinetics and kinematics. Based on previous studies examining vertical jump height [15,16,17,18,19], it was hypothesized that participants would not have differences in jump height following mentally fatiguing tasks. Additionally, given the findings from studies examining physical fatigue effects on vertical jump [13,14], it is speculated that despite maintaining jump heights, mental fatigue would result in changes in the kinematic and kinetic parameters of these jump tasks.

## 2. Materials and Methods

### 2.1. Design

This multi-part investigation involved two studies conducted with different populations. These studies utilized a randomized, within-subject counterbalanced design with single blinding (participant only). For both studies, participants attended two sessions. Each session consisted of participants answering a series of visual analog scales (VAS) prior to and following a cognitive manipulation. Following the VAS, participants performed a set of three individual jumps to assess power and one set of 15 repeated jumps to assess power endurance. These jumps were performed with kinematics being measured via an inertial measurement unit (Study 1) or 3-dimensional motion capture (Study 2), and kinetics being measured with a pair of force platforms. The study protocols were reviewed and approved by the University of Texas at San Antonio Institutional Research Ethics Board (FY21-22-23).

### 2.2. Participants

Study 1 included a convenience sample of 14 collegiate varsity volleyball athletes (See Table 1 for Demographic data). The inclusion criteria for Study 1 specified that participants had to be collegiate varsity volleyball athletes who had not experienced an injury in the past six months and did not experience color blindness. All participants were highly experienced in executing vertical jumps through their regular sport participation.

Study 2 included a convenience sample of 32 recreationally active college students (*n* = 17 females); however, five participants were excluded due to dropping out from the study due to illness or data collection issues, resulting in a total sample of 27 participants (See demographic data on Table 1). For Study 2, based on an a priori power analysis determined based on a medium effect size (*d* = −0.55) from the most recent meta-analysis investigating the effects of mental fatigue on self-control [6], in order to achieve 95% power for a two condition within-group design with alpha set at 0.05, 38 participants were required. The inclusion criteria for Study 2 specified that participants had to currently meet the ACSM guidelines for weekly physical activity engagement (i.e., 150 min of moderate-to-vigorous intensity activity) [20], be between the ages of 18 and 25 years old, not have experienced an injury in the past six-months, and not be experiencing color blindness. Study 2 participants were considered to be recreationally active based on self-reporting experience in vertical jump execution through participation in sports such as basketball and volleyball.

### 2.3. Procedures

**Figure 1:**
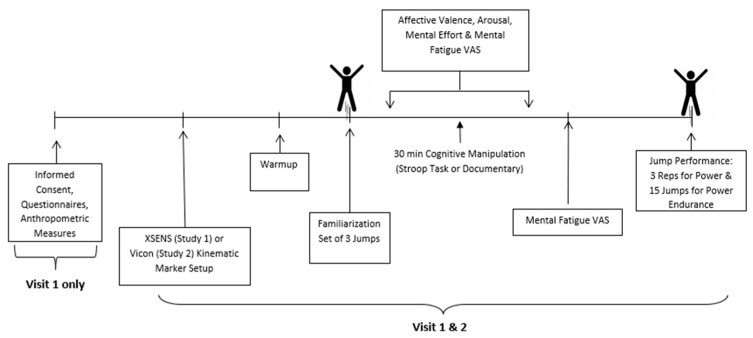
Overview of the study protocols used for these studies.

Study Protocols. Studies 1 and 2 followed identical protocols, differing only in the methods of obtaining kinematic measures during the physical task in Study 2. For Study 1, kinematic measures were obtained with inertial measurement units, and for Study 2, kinematic measures were obtained with three-dimensional motion capture. For both studies, participants were compensated 15 USD for their completion of each session of the study. For illustrative purposes, an overview of the protocol for Study 1 is presented in Figure 1. During the first session, participants provided informed consent and then completed a demographic questionnaire, the Physical Activity Readiness Questionnaire (PAR-Q; [21], and the International Physical Activity Questionnaire Short-Form (IPAQ; [22,23]). This was followed by anthropometric measures being taken and kinematic marker placement.

For Study 1, XSENS inertial measurement units (100 Hz: XSENS Technologies, Henderson, NV, USA) were placed on the lower body and sternum of the participants (described below) to obtain kinematic measures. In Study 2 only, measures of the leg length, knee width, and ankle width were recorded following the VICON Nexus lower body plug-in gait guidelines for kinematic measurement. Furthermore, 16 markers were placed on the participants following the guidelines for the lower body plug-in gait. This was followed by a standardized warm-up consisting of static and dynamic stretches targeting the lower body. Following the warm-up, participants were instructed on how to perform a squat jump and were given a set of three practice jumps as familiarization. Upon completion of these jumps, participants completed a series of measures to assess their mood, arousal, and mental fatigue. Next, participants were randomly assigned to one of the two experimental manipulations (mental fatigue or control) for 30 min, with the alternate manipulation being completed on the final visit. Upon completion of the respective experimental manipulation, participants were asked to complete another series of measures to assess mental demand, task difficulty, mood, arousal, boredom, and mental fatigue, as described in Section 2.4. Participants then completed the task motivation measure and performed the physical tasks, which consisted of three squat jumps as an assessment of maximal power in which they reset their stance after each jump, followed by a set of 15 continuous countermovement jumps to measure power endurance. All jumps were conducted while participants were standing on two force platforms. Participants rated their perceived exertion after each set of jumps. During the entirety of the study, the researchers only interacted with the participants to collect measures and ensure their safety—any form of motivational encouragement was deliberately withheld at all times.

### 2.4. Measures

Demographics and Physical Activity History. Demographics included self-reported sex, and age. Anthropometric data included height and weight, which were obtained using a calibrated Detecto weigh scale (3P7044) and a stadiometer (Seca 213 Portable Measuring Rod, Seca, Hamburg, Germany), which were used to calculate body mass index (mass (kg)/height (m)^2^). The Physical Activity Readiness Questionnaire (PAR-Q) was used to determine whether participants were fit to perform physical activity [21]. The International Physical Activity Questionnaire was used to assess weekly minutes of moderate-to-vigorous physical activity during the past six months [22,23].

### 2.5. Psychological Measures

Mental Demand. Participants rated how mentally demanding each of the experimental manipulations was using the Mental Demand subscale of the National Aeronautics and Space Administration Task Load Index: NASA TLX [24]. The single-item measure is rated on a 20-point scale with bipolar descriptors ranging from (very low) to (very high).

Visual Analog Scales (VASs). The Visual Analog Scales of Boredom, Task Difficulty, and Task Motivation were used [25]. Prior to and following the experimental manipulations, participants were instructed to complete each VAS and were provided instructions of: “Please mark (X) on the line to indicate how you are feeling right now for each of the following items”. The scale consisted of a 100 mm line with anchors ranging from “not at all” on the left side of the line corresponding with 0 to “extremely” on the right side of the line corresponding with 100. The scores for each were calculated by measuring the distance (in mm) from the left side of the scale to where the X was marked.

Mental Fatigue VAS. Mental fatigue was measured using the fatigue and energy subscales from an 18-item VAS [26]. Example items included “tired”, “energetic”, “exhausted”, and “lively”. The fatigue subscale consists of 13 items, whereas the energy subscale consists of five items. Scores were calculated by measuring the distance (in mm) to the ‘X’ from the left side of the scale for each of the 18 items and calculating the average of the 13 fatigue items and five energy items to represent overall changes in fatigue. Internal consistency for the fatigue and energy subscales were deemed good to excellent (fatigue Cronbach’s α = 0.92–0.98; energy Cronbach’s α = 0.83–0.95).

Mood. The Mood Self-Assessment Manikin was used to assess mood prior to and following completion of the experimental manipulation [27]. The scale consists of a cartoon-type figure in which five emotional expressions, ranging from frowning and unhappy to smiling and happy, are represented, on a nine-point scale. Participants were asked to circle the manikin that best represented their current feeling.

Arousal. The Arousal Self-Assessment Manikin was used to assess arousal prior to and following completion of the experimental manipulation [27]. The scale consists of a cartoon-type figure in which five expressions, ranging from calm and relaxed to excited and wide-eyed, are represented, on a nine-point scale. Participants were asked to circle the manikin that best represented their current feeling.

Ratings of Perceived Exertion (RPE). Participants verbally reported their perception of exertion using Borg’s CR-100 RPE Scale [28]. The scale ranged from 0 (no exertion at all) to 100 (maximum perception of exertion). Participants were asked to provide their RPE following the three squat jumps for maximal power and again following the 15 repeated squat jumps.

### 2.6. Physical Performance Measures

Kinetics. For both studies, vertical ground reaction forces for each jump were collected using two AMTI force platforms collecting at 1000 Hz (Advanced Mechanical Technology, Inc., Watertown, MA, USA). Data were collected via VICON Nexus software (2.16, Vicon Motion Systems, Oxford, UK). Force data were summed from the two force platforms and exported to MATLAB where kinetic variables were calculated with a custom MATLAB code. Kinetic variables measured included peak force prior to take off and peak landing force.

Kinematics. For Study 1 only, an XSENS MTw Awinda inertial measurement unit was utilized. Shoe length, hip height, hip width, knee height, and ankle height were measured as per the user guidelines provided in the XSENS inertial measurement unit manual. Participants were fitted with eight sensors following the lower body with sternum configuration described in the XSENS user manual. More specifically, participants had sensors placed on the sternum, pelvis, left and right upper leg on the lateral side above the knee, left and right lower leg on the medial surface of the tibia, and left and right feet on the middle bridge of the foot. All sensors were attached to a Velcro strap and were taped with athletic tape to ensure the sensors did not fall off the participants. Data were collected at a sampling rate of 100 Hz utilizing the XSENS MT software suite v.4.6 (XSENS Technologies, Henderson, NV, USA). Kinematic data were then exported to MATLAB (v2022b, MathWorks, Inc., Natick, MA, USA), where kinematic variables were determined with a custom MATLAB code. Kinematic measures of peak hip flexion, knee flexion, and ankle dorsiflexion prior to jump take off were collected for each jump.

For Study 2 only, recreationally active participants were fitted with 16–14 mm pearl markers. These markers were placed on the left and right anterior superior iliac spine, posterior iliac spine, lateral knee, lateral malleolus of the ankle, calcaneus, over the second metatarsal head on of the foot, thighs, and tibia as per the Vicon plug in gait guidelines. Kinematic data were recorded at 100 Hz on VICON Nexus software (v.2.14, Vicon Motion Systems Ltd., Oxford, UK) using four Vicon Vero cameras (v1.3, Vicon Motion Systems Ltd., Oxford, UK). Data were then exported to MATLAB where kinematic variables were determined with a custom MATLAB code. Kinematic measures of peak hip flexion, knee flexion, and ankle dorsiflexion prior to jump take off were collected for each jump.

### 2.7. Experimental Manipulations

High Cognitive Control Exertion. A computerized version of the Stroop task was used as the mentally fatiguing manipulation [29]. The Stroop task involves response inhibition, a central component of executive function, and is one of the most commonly used manipulations for examining the effects of mental fatigue on physical performance [30,31,32]. Using Inquisit software (Version 6, Millisecond Software, Seattle, WA, USA), three types of stimuli were presented on a white background in either red, blue, black, or green. Stimuli included congruent words (color of word and meaning of word are the same), incongruent words (color of word and meaning of word are different), and control shapes (colored rectangles). Participants completed a total of 900 randomly sampled trials, which consisted of an even balance (*n* = 75) of each of the 12 possible combinations of colors (four: red, blue, black, and green) and color-stimulus congruency (three: congruent, incongruent, and control). Stimuli were presented for up to 2000 ms until a response was recorded, followed by a 200 ms inter-trial interval. Participants were instructed to respond as quickly and accurately as possible to each trial by pressing one of four buttons on the keyboard that corresponded to the color of the font in which the word (or rectangle in the case of control trials) was presented and ignore the printed word (e.g., for the word “black” presented in “red,” they would select the button that corresponded to “red”). This task required participants to inhibit their dominant response of reading the printed word and instead replace it with the subordinate response of identifying the font color. The Stroop task took approximately 30 min to complete. Prior to the commencement of the Stroop task, participants were required to complete a one-minute familiarization, in which individuals were required to achieve a minimum accuracy of 80% before advancing. All participants achieved this target accuracy prior to completing the 30 min Stroop task.

Low Cognitive Control Exertion. Participants watched a 30 min segment of the Fresh Water episode from the Planet Earth documentary series [33]. Documentary films are commonly used as control tasks in studies investigating the effects of mental fatigue on physical performance [31,34,35], as these manipulations have been found to induce no changes in participants’ affective state or arousal, ultimately suggesting that the task is neither relaxing nor boring [35].

### 2.8. Statistical Analysis

For each study, descriptive statistics were computed for all study variables. The effectiveness of our experimental manipulations on mental fatigue was evaluated using a linear mixed effects model with fixed main effects specified for Time (pre-post experimental manipulation) and Condition (mental fatigue vs. control), as well as a Time × Condition interaction, with Subject set as a random effect. Similar linear mixed effects models with Time (pre-post experimental manipulation) × Condition (mental fatigue vs. control) interactions were also computed for mood and arousal. Separate linear mixed effects models with a fixed effect for Condition (mental fatigue vs. control) and a random effect for Subject were computed for mental demand, task difficulty, boredom, task motivation, and RPE.

Separate analyses were computed for our primary outcomes of interest, in that models were computed for the three maximal jumps as well as for the 15 countermovement jumps. The outcomes of interest consisted of jump height, peak concentric force, peak landing force, peak right hip flexion, peak right knee flexion, and peak right ankle dorsiflexion. Both right and left hip flexion, knee flexion, and dorsiflexion were collected, but due to the similarity between the joint angles, only the results for the right side are reported here.

For the three maximal jumps, a series of linear mixed effects models with a fixed effect for Condition (mental fatigue vs. control) and a random effect for Subject were computed for each outcome of interest. For the 15 countermovement jumps, a series of linear mixed effects models with fixed main effects specified for Time (repetitions 1 to 15) and Condition (mental fatigue vs. control), as well as a Time × Condition interaction, and Subject set as a random effect, for all outcomes of interest.

All analyses were performed in R (version 4.3.0) and R Studio (version 2023.06.0, PBC, Boston, MA, USA) using the stats (R Core Team, 2013) and lme4 packages [36]. Linear mixed models were employed as they provide several advantages over repeated measures analysis of variance [37].

Lastly, repeated measures correlations were conducted to examine the associations between the two mental fatigue domains (energy and fatigue domains) and physical performance (i.e., squat jump height and average repeated jump height measures). Significance was set at α = 0.05 for all analysis.

## 3. Results

### 3.1. Psychological Measures

Mental Fatigue—Fatigue Subscale. Descriptives are displayed in Table S1. For Study 1, the results show a significant main effect for Time, indicating greater fatigue following both conditions (see Table 2). However, the main effect for Condition and the Time × Condition interaction were not significant. For Study 2, the results indicate a significant main effect for Time, indicative of greater fatigue following both conditions. However, the main effect for Condition and the Time × Condition interaction were also not significant.

Mental Fatigue—Energy Subscale. For Study 1, the results showed a significant main effect for Time, indicating lower energy following both conditions (See Table 2). However, the main effect for Condition and the Time × Condition interaction were not significant. For Study 2, there was a main effect for Time, indicating lower energy following both conditions. However, the main effect for Condition and Time × Condition interaction were not significant.

Mental Demand. For Study 1, there was a significant main effect for Condition, indicating higher mental demand following the experimental condition (See Table 2). Similarly, for Study 2, there was a significant main effect for Condition, indicating higher mental demand following the experimental condition.

Task Difficulty. For Study 1, there was a significant main effect for Condition, indicating higher task difficulty following the experimental condition (See Table 2). Similarly, for Study 2, there was a main effect for Condition indicating higher task difficulty following the experimental condition.

Boredom. For Study 1, there was no main effect for boredom, indicating no differences following the two conditions (See Table 2). In contrast, for Study 2, the results demonstrated a main effect for Condition, indicating greater boredom following the experimental condition.

Motivation. For Study 1, the results showed no significant Time × Condition interaction for motivation, nor were there significant main effects for Condition or Time (See Table 2). For Study 2, the results show there was a significant main effect for Time, indicating lower motivation following both conditions. However, there was not a main effect for Condition and no significant Time × Condition interaction.

Mood. For Study 1, the results show a significant main effect for Time, indicating a decrease in mood following both conditions (See Table 2). The main effect of Condition was not significant and there was no significant Time × Condition interaction. For Study 2, there was a significant Time × Condition interaction, indicating that mood decreased over time for the control condition. Additionally, the results showed a significant main effect for Time, indicating decreased mood following both conditions. However, the main effect for Condition was not significant.

Arousal. For Study 1, the results show a significant main effect of Time, indicating lower arousal following both conditions (See Table 2). However, the main effect of Condition was not significant, and there was no significant Time × Condition interaction. For Study 2, the results show a significant main effect of Time indicating lower arousal following both conditions. However, the main effect of Condition was not significant and there was no significant Time × Condition interaction.

RPE. For Study 1, the results show no main effect for Condition following the maximal squat jumps and there was no main effect of Condition following the set of repeated jumps (See Table 2). For Study 2, there was no main effect for Condition following the maximal squat jumps and no main effect for Condition following the set of repeated jumps.

### 3.2. Maximal Squat Jump Performance

**Figure 2:**
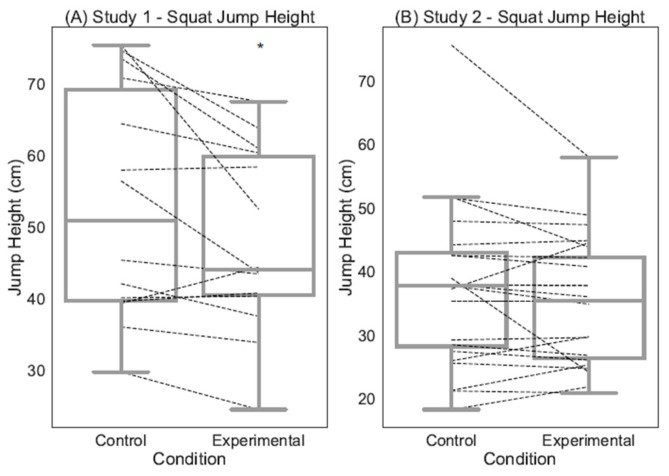
Illustration of the differences in squat jump performance (cm) following each cognitive manipulation condition for Study 1 (**A**) and Study 2 (**B**) at the group level (boxplot) and individual level (dashed lines). * denotes significant a main effect for Condition.

Peak Jump Height. For Study 1, the results show a main effect for Condition, indicating lower jump height following the mental fatigue condition (See Figure 2A). For Study 2, there was not a significant main effect for Condition (See Figure 2B). Statistical test information is presented in Table 3. Descriptives are displayed in Table S2.

Kinematics. For Study 1, the results of our linear mixed effects models reveal no main effect for Condition in right hip flexion, right knee flexion, or right ankle dorsiflexion (See Table 3). Similarly, for Study 2, there was no main effect for Condition in the right hip flexion, right knee flexion, or right ankle dorsiflexion were observed (See Table 3). Descriptives are displayed in Table S2.

Kinetics. For Study 1, the results do not show a significant main effect for Condition for concentric peak force or peak landing force (See Table 3). Similarly, for Study 2, there was no main effect for Condition in concentric peak force or peak landing force (See Table 3). Descriptives are displayed in Table S2.

### 3.3. Repeated Endurance Jump Performance

**Figure 3:**
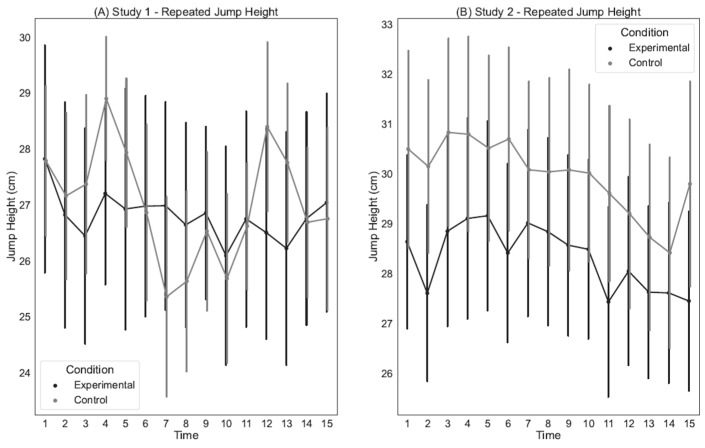
Changes in jump height over the 15 repetitions of the repeated jump test following each cognitive manipulation condition for Study 1 (**A**) and Study 2 (**B**). Bars represent standard error. There was a significant main effect for condition in Study 2 (**B**), but no differences in Study 1 (**A**).

Peak Jump Height. In Study 1, the results show no significant main effects for Time or Condition (See Table 4). There was also no significant Time X Condition interaction (See Figure 3A). For Study 2, the results demonstrate a significant main effect of Condition indicating lower jump height following the mental fatigue condition (See Table 4). There was also a significant main effect for Time, indicating a decrease in jump height over time following both conditions. However, there was not a significant Time × Condition interaction (See Figure 3B). Descriptives for Study 1 are displayed in Table S3, whereas descriptives for Study 2 are presented in Table S4.

Kinematics. For Study 1, the results show no significant main effects for Time or Condition in measures of right hip flexion (See Table 4). There was also no Time × Condition interaction (See Table 4). For the measures of right knee flexion, there was a significant main effect for Condition indicating greater knee flexion following the mental fatigue condition. The main effect of Time and the Time × Condition interaction were non-significant. Finally, for right ankle dorsiflexion, there was a significant main effect observed for Time indicating increased dorsiflexion over time following both conditions. However, the main effect of Condition and the Time × Condition interaction were non-significant. Descriptives are displayed in Table S3.

For Study 2, the results showed no significant main effects of Time or Condition for right hip flexion (See Table 4). There was also no significant Time × Condition interaction (See Table 4). For right knee flexion, there were no significant main effects of Time or Condition as well as no significant Time × Condition interaction. Lastly, for right ankle dorsiflexion, there were no significant main effects of Time or Condition as well as no significant Condition × Time interaction. Descriptives are presented in Table S4.

Kinetics. For Study 1, the results showed no significant main effects for Time or Condition, as well as no significant Time × Condition interaction for concentric peak force (See Table 4). Similarly, there were no main effects for Time or Condition, as well as no significant Time X Condition interaction for peak landing force. Descriptives are displayed in Table S3.

For Study 2, the results for peak concentric force showed a significant Time × Condition interaction for peak concentric force, indicating that values declined to a greater degree over time following the control condition (See Table 4). There was also a main effect for Time, indicating a decrease in concentric force over time following both conditions. There was no main effect for Condition. For peak landings forces, the results show a significant main effect for Time, indicating decreased landing forces over time following both conditions. However, there was no significant main effect for Condition and no significant Time × Condition interaction. Descriptives are presented in Table S4.

## 4. Discussion

The purpose of the present study was to examine the effects of mental fatigue on maximal power jump and repeated jump performance as well as kinetics and kinematics during jump performance. Using two independent samples of collegiate volleyball athletes and recreationally active college students, we observed differential significant impairments in physical performance following exposure to a mentally fatiguing task. Specifically, among the highly trained collegiate volleyball athlete sample, there was decreased performance in only maximal power squat jump performance following exposure to the mental fatigue experimental manipulation, whereas a similar detrimental effect of mental fatigue was only observed for repeated jump performance among the recreationally active sample. Alterations in movement kinematics were only observed when the collegiate volleyball sample performed the set of repeated jumps, which may have attenuated mental fatigue-induced performance declines. Taken together, these differing findings suggest that mental fatigue has a detrimental impact on anaerobic task performance, which contrasts with the null effect shown in recent studies [15,16,17,18,19], but the present effects appear to be task-dependent and based on an individual’s training status.

To date, very few studies have examined the influence of mental fatigue on anaerobic task performance, with even fewer studies examining variations of vertical jump performance specifically [17,19,38]. Across this body of evidence, findings have generally shown that mental fatigue does not affect countermovement jump performance among both recreationally active participants and samples of trained amateur/collegiate athletes [17,19,38], which partially contradicts the pattern of results across our two studies. Some researchers have suggested that tasks such as vertical jumps, which involve high-intensity exertion over a brief duration, utilize part of the brain (i.e., the posterior cingulate cortex) that is not affected by mental fatigue, which is understood to affect the prefrontal cortex and the dorsolateral prefrontal cortex [17]. Alternatively, other researchers have suggested that the negative effects of mental fatigue only manifest during more complex tasks of prolonged duration [19,38]. While these hypotheses were supported by the null effects observed among the recreationally active sample in the current study, the current findings for the collegiate volleyball athlete sample oppose this line of thought. Differences in sample size and characteristics are among the potential explanations that may explain why these findings for maximal power jump performance among trained athletes did not align with previous research and the effects observed in Study 2. First, the sample of collegiate volleyball athletes was half the size of other studies of amateur/collegiate athletes [19,38] as well as our recreationally active sample in Study 2, and therefore our lack of statistical power in Study 1 may have resulted in a significant difference being observed by chance. Second, previous studies have involved only male athletes, whereas our collegiate volleyball sample was predominantly female, and sex-based differences in susceptibility to mental fatigue may exist [39]. Third, previous studies have found that athletes and non-athletes display different reaction time responses to mental fatigue, with Martin and colleagues suggesting that professional cyclists display better inhibitory control than recreationally active individuals [8] and Jaydari Fard and colleagues suggesting that athletes and non-athletes employ different cognitive strategies resulting in differing reaction time responses to mental fatigue [7]. These conflicting findings point to the continued need to conduct well-powered studies between athletes and non-athletes in addition to the importance of recruiting sex-balanced samples for the purpose of determining whether biological differences may underly the relationship between mental fatigue and physical performance.

The present study represents the first to examine whether mental fatigue affects anaerobic endurance performance. Repeated jump tests have been utilized to examine anaerobic power endurance and how individuals are able to utilize the stretch shortening cycle to perform repeated explosive movements under fatiguing conditions [13]. Similar to the maximal power squat jump task, findings differed across our two samples. For this performance outcome, we observed significantly lower jump heights following the mentally fatiguing condition only among the recreationally active sample, whereas no between-condition differences were observed for the collegiate volleyball athlete sample. A potential reason for the differences in findings between the two groups may be due to training status, as the collegiate volleyball players were able to alter their jump mechanics by increasing knee flexion measures to maintain similar jump heights following the mentally fatiguing conditions, whereas no alterations in jump mechanics were observed among the recreationally active group. Another potential explanation also relates to training status—previous work has shown that trained athletes are more resistant to the negative effects of mental fatigue on endurance performance tasks [8]. Martin and colleagues [8] hypothesized that athletes may be more resilient in the face of mentally fatiguing conditions due to training-induced adaptations that help them to develop better inhibitory control due to increased neuronal efficiency. Future studies seeking to identify the mechanisms by which trained individuals develop better tolerance to mental fatigue are evidently warranted.

This study was also the first to implement kinetic and kinematic measures to determine whether movement alterations underly previously observed fatigue-induced impairments in physical performance. The maximal power and endurance jump tasks were selected as they represented a pragmatic means to study biomechanical alterations in a restricted plane of motion, which also allowed us to capture kinetic forces by having the participants perform the jumps on two force plates. For the maximal power assessment, there were no differences in kinetics or kinematic measures observed. Martin and colleagues [19] found no differences in concentric peak force production in recreationally active participants measured by a linear encoder following a mentally fatiguing condition similar to the findings of the current study. Similarly, it has been shown that following physically fatiguing conditions, neither take off nor landing peak forces were altered [40]. In contrast to the current findings, it has been shown that physically fatiguing tasks do affect hip, knee, and ankle kinematics in countermovement jumps [40]. However, this may be due to the type of jump utilized in the current study. In terms of the repeated jump task, this is the first study to examine how mental fatigue affects power endurance. Physical fatigue during a repeated jump task has been shown to result in decreases in peak force [13], similar to the findings of the current study. Interestingly, for the recreationally active population, despite having higher jumps during the control condition there was a greater decrease in force production than in the mentally fatiguing condition. While it may be expected that lower force production results in lower jump height, this was not the case. Thus, more work is needed to improve our understanding of why this occurred. In terms of kinematics, changes in knee and ankle dorsiflexion have been observed [13] which is partially supported by the current findings, as this was observed in the volleyball athlete population but not in the recreationally active population. Again, the difference in findings may be attributed to differences in training status between populations.

Despite the fatigue-induced impairments that were observed across the jump performance tasks, it is interesting to note that significant between-condition differences were not observed for the pre-performance measures of fatigue and energy. Previous studies have generally used single item measures to assess mental fatigue using visual analog scales and have shown greater mental fatigue following exposure to tasks with greater cognitive control demands [41,42,43]; however, we elected to use Lee’s [26] 18-item fatigue scale to reduce potential measurement error. One shortcoming of this 18-item fatigue instrument, however, is that it is not specific to mental fatigue, as it refers to the construct of fatigue more broadly. From this perspective, it may not be surprising that we observed significant increases in fatigue and decreases in energy with time on task for both experimental manipulations over the 30 min window, as participants were not cued to consider fatigue within a specific domain (i.e., mental). Nevertheless, the results from our other manipulation checks provide confidence that our experimental manipulations were effective. Specifically, following the Stroop task—which involved higher cognitive control demands compared to watching a documentary—we observed significantly greater reports of mental demand and task difficulty. Additionally, boredom was similar across conditions. It is possible that boredom also contributed to elevated scores for fatigue following the control condition by means of task disengagement [44]. Task disengagement can occur when an individual performs a non-engaging or repetitive task for a prolonged period of time and has been found to correspond with greater perceptions of fatigue [44]. While great strides are being made regarding the role of boredom in goal-directed sport and exercise performance [45,46], more work is needed to improve our current understanding of how boredom and fatigue develop with extended exposure to tasks involving high and low cognitive control demands.

Despite several strengths, the current study is not without its limitations. First, as noted earlier, the instrument selected to evaluate perceptions of mental fatigue may not have been too generic to capture the subtle nuance in reporting domain-specific aspects of mental fatigue. Second, our experimental manipulations do not reflect the real-world conditions that may cause an individual to experience heightened perceptions of mental fatigue, and therefore more ecologically valid protocols should be tested. Finally, Study 1 involved a small sample consisting of 12 participants and we did not reach our intended sample size goal for Study 2, which could result in potential type 1 (Study 2) and type 2 (Study 1) errors. The small pool of collegiate volleyball athletes limited our potential sample size for Study 1, which motivated us to conduct a more well-powered study with a general sample (Study 2). Although we achieved 71% of our sample size goal of 38 for Study 2, it is worthwhile to recognize that our sample size of 27 participants represents the largest sample used to investigate the influence of mental fatigue on anaerobic performance to date [17,19].

## 5. Conclusions

This study sought to examine how mental fatigue affects anaerobic performance as well as mechanics in squat jump and repeated jump tasks. Despite the limitations of small sample sizes and lack of real-world scenarios, we found that the mentally fatiguing condition resulted in lower squat jump performance for the collegiate volleyball athletes (Study 1) but not for the recreationally active participants (Study 2). Conversely, for the repeated jump task, the mentally fatiguing condition resulted in worse repeated jump performance for the recreationally active participants but not for the collegiate volleyball athletes, though this may be attributable to alterations in kinematic measures for the volleyball athletes. Overall, these findings suggest that mental fatigue affects collegiate volleyball players and recreationally active individuals differently. Additionally, mental fatigue may have different effects depending on the anaerobic task performed. Therefore, factors such as training status and type of task should be taken into consideration when determining the role that mental fatigue plays in athletic performance.

## Figures and Tables

**Table 1:** Demographics of participants for each study.

	Age	Height (cm)	Weight (kg)	BMI
Study 1 (*n* = 14)	19.71 ± 1.38	175.11 ± 7.58	72.76 ± 7.00	23.78 ± 2.33
Study 2 (*n* = 27)	20.19 ± 1.84	167.83 ± 10.31	70.56 ± 18.90	24.95 ± 6.04

**Table 2:** Psychological variables model results for Study 1 and Study 2.

	Study 1		Study 2	
	Β ± SE	*p*	Β ± SE	*p*
Mental Fatigue—Fatigue Subscale				
Main Effect: Time	18.23 ± 4.63	<0.001	18.00 ± 3.92	<0.001
Main Effect: Condition	−9.63 ± 10.35	0.36	−4.33 ± 8.75	0.62
Interaction: Time × Condition	8.55 ± 6.54	0.20	6.23 ± 5.54	0.26
Mental Fatigue—Energy Subscale				
Main Effect: Time	−14.80 ± 3.91	<0.001	−15.50 ± 4.19	<0.001
Main Effect: Condition	6.26 ± 8.75	0.48	−0.67 ± 9.36	0.99
Interaction: Time × Condition	−5.01 ± 5.53	0.37	−3.92 ± 5.92	0.51
Motivation				
Main Effect: Time	−10.71 ± 5.83	0.07	−20.59 ± 5.74	<0.001
Main Effect: Condition	12.57 ± 13.04	0.96	6.78 ± 12.84	0.60
Interaction: Time × Condition	−11.14 ± 8.25	0.18	−4.22 ± 8.12	0.60
Mood				
Main Effect: Time	−0.86 ± 0.31	<0.01	−2.04 ± 0.30	<0.001
Main Effect: Condition	0.79 ± 0.68	0.26	−1.07 ± 0.67	0.11
Interaction: Time × Condition	−0.79 ± 0.43	0.08	0.89 ± 0.42	0.04
Arousal				
Main Effect: Time	−1.14 ± 0.45	0.01	−1.26 ± 0.32	<0.001
Main Effect: Condition	0.79 ± 1.00	0.44	−0.67 ± 0.71	0.35
Interaction: Time × Condition	−0.43 ± 0.63	0.50	0.65 ± 0.45	0.15
Mental Demand				
Main Effect: Condition	8.46 ± 1.14	<0.001	7.09 ± 1.06	<0.001
Task Difficulty				
Main Effect: Condition	41.14 ± 4.95	<0.001	28.63 ± 4.93	<0.001
Boredom				
Main Effect: Condition	15.36 ± 8.37	0.09	25.93 ± 6.34	<0.001
Perceived Exertion				
Squat Jump Main Effect: Condition	2.79 ± 4.70	0.56	0.67 ± 2.82	0.82
Repeated Jump Main Effect: Condition	4.21 ± 4.37	0.35	2.39 ± 2.35	0.32

**Table 3:** Maximal squat jump performance model results for physical performance, kinematic, and kinetic variables for Study 1 and Study 2.

	Study 1		Study 2	
	Β ± SE	*p*	Β ± SE	*p*
Jump Height (cm)				
Main Effect: Condition	−5.27 ± 1.88	0.01	−2.07 ± 1.08	0.07
Right Hip Flexion (degrees)				
Main Effect: Condition	−2.88 ± 5.47	0.61	1.74 ± 1.93	0.37
Right Knee Flexion (degrees)				
Main Effect: Condition	6.36 ± 4.29	0.16	6.74 ± 4.59	0.16
Right Ankle Dorsi-flexion (degrees)				
Main Effect: Condition	1.76 ± 3.85	0.65	−3.16 ± 2.96	0.29
Concentric Peak Force (N)				
Main Effect: Condition	−15.50 ± 14.34	0.30	−20.34 ± 16.94	0.24
Peak Landing Force (N)				
Main Effect: Condition	−79.26 ± 66.73	0.26	137.17 ± 100.01	0.18

**Table 4:** Repeated jump performance model results for physical performance, kinematic, and kinetic variables for Study 1 and Study 2.

	Study 1		Study 2	
	Β ± SE	*p*	Β ± SE	*p*
Jump Height (cm)				
Main Effect: Time	−0.06 ± 0.05	0.22	−0.13 ± 0.03	<0.001
Main Effect: Condition	−0.38 ± 2.12	0.86	−1.12 ± 0.43	<0.01
Interaction: Time × Condition	0.02 ± 0.07	0.73	0.05 ± 0.05	0.27
Right Hip Flexion (degrees)				
Main Effect: Time	−0.14 ± 0.24	0.55	0.05 ± 0.10	0.60
Main Effect: Condition	5.62 ± 3.04	0.07	−0.19 ± 0.97	0.85
Interaction: Time × Condition	−0.47 ± 0.34	0.16	0.18 ± 0.11	0.10
Right Knee Flexion (degrees)				
Main Effect: Time	0.13 ± 0.16	0.41	0.07 ± 0.15	0.65
Main Effect: Condition	5.32 ± 2.02	<0.01	0.99 ± 2.01	0.62
Interaction: Time × Condition	−0.29 ± 0.22	0.19	−0.18 ± 0.22	0.41
Right Ankle Dorsi-flexion (degrees)				
Main Effect: Time	0.14 ± 0.04	<0.01	0.03 ± 0.05	0.55
Main Effect: Condition	−0.84 ± 2.99	0.78	0.05 ± 0.67	0.94
Interaction: Time × Condition	−0.03 ± 0.06	0.65	−0.04 ± 0.07	0.56
Concentric Peak Force (N)				
Main Effect: Time	0.79 ± 9.22	0.93	−5.57 ± 2.50	0.03
Main Effect: Condition	20.15 ± 76.08	0.79	−16.16 ± 19.74	0.41
Interaction: Time × Condition	12.71 ± 8.40	0.13	5.65 ± 2.16	<0.001
Peak Landing Force (N)				
Main Effect: Time	−1.80 ± 6.45	0.78	−10.62 ± 4.47	0.02
Main Effect: Condition	145.79 ± 82.57	0.08	−111.71 ± 153.61	0.05
Interaction: Time × Condition	1.84 ± 9.12	0.84	11.83 ± 6.25	0.06

## Data Availability

Data are available upon request.

## Supplementary Materials

The following supporting information can be downloaded at: https://www.mdpi.com/article/10.3390/sports12070192/s1, Table S1: Illustrates the mean ± standard error of the psychological measures from Study 1 and Study 2 as well as differences in these measures following each of the cognitive manipulations; Table S2: Illustrates the mean ± standard error of Squat Jump Measures for Study 1 and Study 2 as well as differences in jump measures following each cognitive manipulation; Table S3: Descriptive measures of the repeated jump measures for the volleyball athletes in Study 1 displayed as Means ± standard error; Table S4: Descriptive measure of the repeated jump measures for the recreationally active population in Study 2 displayed as Means ± standard error.
