# Supplementary material for: The Effects of Mental Fatigue on Anaerobic Power and Power Endurance Performance
Source: Sports (Basel). 2024 Jul 16;12(7):192. doi: 10.3390/sports12070192 (PMC11280764; doi:10.3390/sports12070192)
Supplement: Supplementary file 1 [file sports-12-00192-s001.zip › sports-3074034-supplementary.pdf]

**Table S1.** Illustrates the mean ± standard error of the psychological measures from Study 1 and Study 2 as well as differences in these measures following each of the cognitive manipulations.

Study 1 – Volleyball Athletes

| | Motivation | | Mood | | Mental Fatigue- Fatigue Subscale | | Mental Fatigue– Energy Subscale | | Arousal | | Mental Demand | Task Difficulty | Boredom |
|---|---|---|---|---|---|---|---|---|---|---|---|---|---|
| | Pre | Post | Pre | Post | Pre | Post | Pre | Post | Pre | Post | | | |
| Exp | 66.57 ± 4.70 | 44.71 ± 7.41 | 6.93 ± 0.20 | 5.29 ± 0.41# | 21.18 ± 4.26 | 47.96 ± 7.31# | 55.70 ± 4.55 | 35.89 ± 5.85# | 4.36 ± 0.51 | 2.79 ± 0.56# | 16.81 ± 0.44* | 61.21 ± 5.13* | 66.50 ± 7.05 |
| Control | 65.14 ± 6.04 | 54.43 ± 5.32 | 6.93 ± 0.29 | 6.07 ± 0.30# | 22.25 ± 4.85 | 40.49 ± 7.45# | 54.46 ± 4.60 | 39.66 ± 5.13# | 4.00 ± 0.55 | 2.86 ± 0.55# | 8.32 ± 1.11 | 20.07 ± 5.33 | 51.14 ± 8.17 |

Study 2 – Recreationally Active Students

| | Motivation | | Mood | | Mental Fatigue - Fatigue Subscale | | Mental Fatigue– Energy Subscale | | Arousal | | Mental Demand | Task Difficulty | Boredom |
|---|---|---|---|---|---|---|---|---|---|---|---|---|---|
| | Pre | Post | Pre | Post | Pre | Post | Pre | Post | Pre | Post | | | |
| Exp | 72.30 ± 3.88 | 51.70 ± 5.25# | 7.15 ± 0.21 | 5.11 ± 0.30# | 17.40 ± 2.85 | 41.63 ± 4.36# | 56.43 ± 3.17 | 37.01 ± 4.34# | 3.74 ± 0.40 | 2.48 ± 0.33# | 12.52 ± 0.90* | 47.07 ± 4.82* | 71.63 ± 4.06* |
| Control | 74.85 ± 3.51 | 50.04 ± 4.96# | 6.96 ± 0.21 | 5.81 ± 0.29# | 15.50 ± 2.30 | 33.50 ± 4.19# | 60.41 ± 3.84 | 44.91 ± 4.55# | 3.72 ± 0.41 | 3.11 ± 0.38# | 5.43 ± 0.90 | 18.44 ± 4.29 | 45.70 ± 5.03 |

* = significantly different between conditions; # = significantly different from pre-cognitive measures; exp = experimental condition.

**Table S2.** Illustrates the mean ± standard error of Squat Jump Measures for Study 1 and Study 2 as well as differences in jump measures following each cognitive manipulation.

| | Jump Height (cm) | Right Hip Flexion (degrees) | Right Knee Flexion (degrees) | Right Ankle Dorsiflexion (degrees) | Concentric Peak Force (N) | Peak Landing Force (N) |
|---|---|---|---|---|---|---|
| **Volleyball Athletes** | | | | | | |
| Exp. | 47.99 ± 3.40* | 47.87 ± 4.86 | 55.80 ± 2.10 | 36.57 ± 5.26 | 1544.07 ± 60.53 | 2568.56 ± 141.60 |
| Control | 53.26 ± 4.34 | 52.75 ± 4.88 | 49.44 ± 4.78 | 34.81 ± 5.41 | 1573.66 ± 57.45 | 2690.99 ± 143.34 |
| **Recreationally Active** | | | | | | |
| | Jump Height (cm) | Right Hip Flexion (degrees) | Right Knee Flexion (degrees) | Right Ankle Dorsiflexion (degrees) | Concentric Peak Force (N) | Peak Landing Force (N) |
| Exp. | 35.04 ± 1.85 | 17.46 ± 1.65 | 41.92 ± 4.77 | 6.21 ± 0.63 | 1387.03 ± 87.42 | 2653.67 ± 185.39 |
| Control | 37.23 ± 2.40 | 16.11 ± 1.61 | 36.42 ± 4.35 | 9.25 ± 3.04 | 1450.13 ± 92.78 | 2670.30 ± 164.26 |

* = significantly different between conditions; exp = experimental.

**Table S3.** Descriptive measures of the repeated jump measures for the volleyball athletes in Study 1 displayed as Means ± standard error.

| | **Jump Height 1 (cm)** | **Jump Height 2 (cm)** | **Jump Height 3 (cm)** | **Jump Height 4 (cm)** | **Jump Height 5 (cm)** | **Jump Height 6 (cm)** | **Jump Height 7 (cm)** | **Jump Height 8 (cm)** | **Jump Height 9 (cm)** | **Jump Height 10 (cm)** | **Jump Height 11 (cm)** | **Jump Height 12 (cm)** | **Jump Height 13 (cm)** | **Jump Height 14 (cm)** | **Jump Height 15 (cm)** |
|---|---|---|---|---|---|---|---|---|---|---|---|---|---|---|---|
| **Exp.** | 27.82 ± 2.03 | 26.82 ± 2.02 | 26.45 ± 1.93 | 27.20 ± 1.62 | 26.93 ± 2.16 | 26.98 ± 1.97 | 26.99 ± 1.86 | 26.64 ± 1.82 | 26.86 ± 1.54 | 26.09 ± 1.95 | 26.75 ± 1.93 | 26.51 ± 1.90 | 26.23 ± 2.08 | 26.76 ± 1.91 | 27.04 ± 1.95 |
| **Control** | 27.79 ± 1.34 | 27.16 ± 1.49 | 27.37 ± 1.60 | 28.90 ± 1.11 | 27.94 ± 1.33 | 26.86 ± 1.57 | 25.36 ± 1.79 | 25.64 ± 1.61 | 26.53 ± 1.42 | 25.69 ± 1.51 | 26.62 ± 1.13 | 28.40 ± 1.51 | 27.75 ± 1.42 | 26.69 ± 1.34 | 26.75 ± 1.63 |
| | **Right Hip Flexion (deg) 1** | **Right Hip Flexion (deg) 2** | **Right Hip Flexion (deg) 3** | **Right Hip Flexion (deg) 4** | **Right Hip Flexion (deg) 5** | **Right Hip Flexion (deg) 6** | **Right Hip Flexion (deg) 7** | **Right Hip Flexion (deg) 8** | **Right Hip Flexion (deg) 9** | **Right Hip Flexion (deg) 10** | **Right Hip Flexion (deg) 11** | **Right Hip Flexion (deg) 12** | **Right Hip Flexion (deg) 13** | **Right Hip Flexion (deg) 14** | **Right Hip Flexion (deg) 15** |
| **Exp.** | 98.23 ± 5.59 | 91.20 ± 6.67 | 90.73 ± 7.48 | 90.37 ± 7.64 | 89.12 ± 7.78 | 89.28 ± 8.17 | 88.52 ± 8.45 | 88.99 ± 8.43 | 86.95 ± 8.36 | 89.65 ± 8.22 | 87.12 ± 8.94 | 87.07 ± 8.89 | 88.09 ± 8.85 | 88.51 ± 8.24 | 82.64 ± 7.54 |
| **Control** | 89.84 ± 6.25 | 87.64 ± 6.79 | 87.52 ± 7.45 | 87.49 ± 7.27 | 86.40 ± 7.68 | 86.84 ± 7.47 | 87.15 ± 8.01 | 83.54 ± 7.26 | 89.84 ± 7.88 | 89.16 ± 8.09 | 87.70 ± 8.52 | 85.96 ± 7.62 | 85.49 ± 8.06 | 86.13 ± 8.53 | 86.62 ± 9.08 |
| | **Right Knee Flexion (deg) 1** | **Right Knee Flexion (deg) 2** | **Right Knee Flexion (deg) 3** | **Right Knee Flexion (deg) 4** | **Right Knee Flexion (deg) 5** | **Right Knee Flexion (deg) 6** | **Right Knee Flexion (deg) 7** | **Right Knee Flexion (deg) 8** | **Right Knee Flexion (deg) 9** | **Right Knee Flexion (deg) 10** | **Right Knee Flexion (deg) 11** | **Right Knee Flexion (deg) 12** | **Right Knee Flexion (deg) 13** | **Right Knee Flexion (deg) 14** | **Right Knee Flexion (deg) 15** |
| **Exp.** | 78.62 ± 3.71 | 76.47 ± 3.67 | 77.61 ± 3.39 | 78.00 ± 4.26 | 75.37 ± 3.60 | 76.28 ± 3.64 | 75.05 ± 3.69 | 75.59 ± 4.19 | 74.58 ± 4.18 | 76.46 ± 4.37 | 76.21 ± 4.63 | 77.16 ± 4.84 | 76.93 ± 4.63 | 77.02 ± 4.47 | 73.08 ± 4.46 |
| **Control** | 71.90 ± 4.47 | 72.31 ± 4.93 | 72.70 ± 4.82 | 73.87 ± 4.39 | 73.07 ± 5.15 | 73.12 ± 4.98 | 72.82 ± 5.17 | 73.35 ± 4.93 | 72.94 ± 5.19 | 73.33 ± 5.19 | 74.07 ± 5.06 | 73.08 ± 4.92 | 73.64 ± 4.01 | 73.67 ± 4.39 | 74.71 ± 5.14 |
| | **Right Ankle Dorsiflexion (deg) 1** | **Right Ankle Dorsiflexion (deg) 2** | **Right Ankle Dorsiflexion (deg) 3** | **Right Ankle Dorsiflexion (deg) 4** | **Right Ankle Dorsiflexion (deg) 5** | **Right Ankle Dorsiflexion (deg) 6** | **Right Ankle Dorsiflexion (deg) 7** | **Right Ankle Dorsiflexion (deg) 8** | **Right Ankle Dorsiflexion (deg) 9** | **Right Ankle Dorsiflexion (deg) 10** | **Right Ankle Dorsiflexion (deg) 11** | **Right Ankle Dorsiflexion (deg) 12** | **Right Ankle Dorsiflexion (deg) 13** | **Right Ankle Dorsiflexion (deg) 14** | **Right Ankle Dorsiflexion (deg) 15** |
| **Exp.** | 23.20 ± 1.25 | 23.40 ± 1.90 | 23.64 ± 1.84 | 25.10 ± 1.41 | 24.07 ± 1.92 | 24.03 ± 1.66 | 24.10 ± 1.41 | 24.30 ± 1.73 | 24.38 ± 1.41 | 24.19 ± 1.63 | 23.90 ± 1.62 | 25.01 ± 1.47 | 24.76 ± 1.35 | 24.86 ± 1.64 | 25.76 ± 1.65 |
| **Control** | 24.06 ± 2.45 | 24.06 ± 3.04 | 24.70 ± 2.91 | 25.41 ± 2.80 | 25.37 ± 2.95 | 25.28 ± 2.49 | 25.57 ± 2.68 | 25.71 ± 2.85 | 24.41 ± 2.55 | 25.38 ± 2.51 | 25.75 ± 2.58 | 25.91 ± 2.73 | 26.19 ± 3.12 | 26.97 ± 3.45 | 25.43 ± 3.12 |
| | **Peak Concentric Force (N) 1** | **Peak Concentric Force (N) 2** | **Peak Concentric Force (N) 3** | **Peak Concentric Force (N) 4** | **Peak Concentric Force (N) 5** | **Peak Concentric Force (N) 6** | **Peak Concentric Force (N) 7** | **Peak Concentric Force (N) 8** | **Peak Concentric Force (N) 9** | **Peak Concentric Force (N) 10** | **Peak Concentric Force (N) 11** | **Peak Concentric Force (N) 12** | **Peak Concentric Force (N) 13** | **Peak Concentric Force (N) 14** | **Peak Concentric Force (N) 15** |
| **Exp.** | 1655.45 ± 85.40 | 2296.79 ± 154.87 | 1746.94 ± 109.88 | 2224.44 ± 167.68 | 1697.81 ± 101.61 | 2098.19 ± 133.84 | 1837.34 ± 122.83 | 2158.99 ± 136.28 | 1896.38 ± 131.65 | 2239.57 ± 196.25 | 1917.22 ± 164.71 | 2204.65 ± 169.06 | 1929.74 ± 167.30 | 2261.88 ± 207.06 | 1929.08 ± 217.95 |
| **Control** | 1702.43 ± 78.14 | 2221.10 ± 114.53 | 1714.00 ± 87.95 | 1996.33 ± 100.87 | 1687.87 ± 70.10 | 1871.73 ± 95.31 | 1731.40 ± 98.99 | 2218.42 ± 156.71 | 1755.30 ± 112.52 | 2049.64 ± 91.10 | 1785.35 ± 118.53 | 2064.80 ± 146.25 | 1718.17 ± 113.71 | 1993.10 ± 134.65 | 1760.07 ± 134.17 |
| | **Peak Landing Force (N) 1** | **Peak Landing Force (N) 2** | **Peak Landing Force (N) 3** | **Peak Landing Force (N) 4** | **Peak Landing Force (N) 5** | **Peak Landing Force (N) 6** | **Peak Landing Force (N) 7** | **Peak Landing Force (N) 8** | **Peak Landing Force (N) 9** | **Peak Landing Force (N) 10** | **Peak Landing Force (N) 11** | **Peak Landing Force (N) 12** | **Peak Landing Force (N) 13** | **Peak Landing Force (N) 14** | **Peak Landing Force (N) 15** |
| **Exp.** | 2135.54 ± 191.78 | 1920.63 ± 177.97 | 2191.09 ± 166.32 | 1920.58 ± 187.60 | 2209.66 ± 234.17 | 1942.34 ± 202.85 | 2285.33 ± 214.39 | 1906.96 ± 200.05 | 2187.02 ± 221.59 | 1957.71 ± 230.00 | 2229.70 ± 201.63 | 1912.41 ± 201.28 | 2156.46 ± 175.09 | 1996.01 ± 204.97 | 2115.37 ± 137.18 |
| **Control** | 2216.98 ± 168.70 | 1724.24 ± 104.81 | 2015.68 ± 155.58 | 1751.90 ± 123.66 | 2026.82 ± 129.84 | 1738.65 ± 109.79 | 2054.86 ± 136.03 | 1729.49 ± 111.99 | 1955.09 ± 131.08 | 1771.11 ± 115.76 | 2025.67 ± 156.76 | 1749.65 ± 115.04 | 1977.53 ± 145.51 | 1763.56 ± 127.47 | 2174.06 ± 163.20 |

Exp. = experimental; deg = degrees.

**Table S4.** Descriptive measure of the repeated jump measures for the recreationally active population in Study 2 displayed as Means ± standard error.

| | **Jump Height 1 (cm)** | **Jump Height 2 (cm)** | **Jump Height 3 (cm)** | **Jump Height 4 (cm)** | **Jump Height 5 (cm)** | **Jump Height 6 (cm)** | **Jump Height 7 (cm)** | **Jump Height 8 (cm)** | **Jump Height 9 (cm)** | **Jump Height 10 (cm)** | **Jump Height 11 (cm)** | **Jump Height 12 (cm)** | **Jump Height 13 (cm)** | **Jump Height 14 (cm)** | **Jump Height 15 (cm)** |
|---|---|---|---|---|---|---|---|---|---|---|---|---|---|---|---|
| **Exp.** | 28.64 ± 1.74 | 27.61 ± 1.77 | 28.85 ± 1.90 | 29.10 ± 2.01 | 29.16 ± 1.90 | 28.41 ± 1.79 | 29.01 ± 1.87 | 28.84 ± 1.88 | 28.57 ± 1.81 | 28.49 ± 1.80 | 27.43 ± 1.90 | 28.05 ± 1.89 | 27.63 ± 1.73 | 27.61 ± 1.81 | 27.45 ± 1.80 |
| **Control** | 30.50 ± 1.97 | 30.15 ± 1.73 | 30.83 ± 1.89 | 30.79 ± 1.95 | 30.51 ± 1.86 | 30.70 ± 1.84 | 30.08 ± 1.77 | 30.04 ± 1.88 | 30.08 ± 2.02 | 30.01 ± 1.78 | 29.60 ± 1.77 | 29.20 ± 1.90 | 28.73 ± 1.86 | 28.42 ± 1.91 | 29.80 ± 2.06 |
| | **Right Hip Flexion (deg) 1** | **Right Hip Flexion (deg) 2** | **Right Hip Flexion (deg) 3** | **Right Hip Flexion (deg) 4** | **Right Hip Flexion (deg) 5** | **Right Hip Flexion (deg) 6** | **Right Hip Flexion (deg) 7** | **Right Hip Flexion (deg) 8** | **Right Hip Flexion (deg) 9** | **Right Hip Flexion (deg) 10** | **Right Hip Flexion (deg) 11** | **Right Hip Flexion (deg) 12** | **Right Hip Flexion (deg) 13** | **Right Hip Flexion (deg) 14** | **Right Hip Flexion (deg) 15** |
| **Exp.** | 13.69 ± 1.65 | 13.72 ± 1.62 | 15.84 ± 1.81 | 15.31 ± 2.01 | 16.68 ± 2.16 | 16.86 ± 2.21 | 15.58 ± 2.11 | 18.02 ± 2.73 | 16.42 ± 2.35 | 16.93 ± 2.01 | 15.63 ± 2.21 | 15.96 ± 2.63 | 17.14 ± 2.49 | 16.36 ± 2.33 | 16.68 ± 3.34 |
| **Control** | 13.24 ± 1.55 | 15.91 ± 2.19 | 17.00 ± 2.38 | 16.23 ± 2.13 | 15.69 ± 2.23 | 17.00 ± 2.02 | 15.56 ± 2.45 | 15.24 ± 2.26 | 14.74 ± 2.24 | 15.86 ± 2.28 | 17.04 ± 2.21 | 16.94 ± 2.47 | 15.39 ± 2.16 | 16.09 ± 2.04 | 15.85 ± 1.95 |
| | **Right Knee Flexion (deg) 1** | **Right Knee Flexion (deg) 2** | **Right Knee Flexion (deg) 3** | **Right Knee Flexion (deg) 4** | **Right Knee Flexion (deg) 5** | **Right Knee Flexion (deg) 6** | **Right Knee Flexion (deg) 7** | **Right Knee Flexion (deg) 8** | **Right Knee Flexion (deg) 9** | **Right Knee Flexion (deg) 10** | **Right Knee Flexion (deg) 11** | **Right Knee Flexion (deg) 12** | **Right Knee Flexion (deg) 13** | **Right Knee Flexion (deg) 14** | **Right Knee Flexion (deg) 15** |
| **Exp.** | 30.16 ± 3.92 | 34.90 ± 3.60 | 34.32 ± 4.16 | 35.10 ± 4.07 | 32.67 ± 4.29 | 32.33 ± 3.96 | 31.20 ± 3.95 | 32.43 ± 4.29 | 32.09 ± 3.90 | 31.41 ± 3.94 | 32.04 ± 3.98 | 33.76 ± 4.24 | 31.49 ± 4.25 | 30.59 ± 4.16 | 33.62 ± 4.23 |
| **Control** | 36.10 ± 5.36 | 36.22 ± 4.67 | 37.74 ± 4.96 | 39.06 ± 5.02 | 38.77 ± 4.86 | 40.58 ± 5.06 | 40.11 ± 4.99 | 38.95 ± 4.82 | 40.26 ± 5.19 | 37.71 ± 4.13 | 40.75 ± 5.06 | 39.01 ± 5.18 | 37.77 ± 4.33 | 36.89 ± 4.99 | 38.32 ± 4.65 |
| | **Right Ankle Dorsiflexion (deg) 1** | **Right Ankle Dorsiflexion (deg) 2** | **Right Ankle Dorsiflexion (deg) 3** | **Right Ankle Dorsiflexion (deg) 4** | **Right Ankle Dorsiflexion (deg) 5** | **Right Ankle Dorsiflexion (deg) 6** | **Right Ankle Dorsiflexion (deg) 7** | **Right Ankle Dorsiflexion (deg) 8** | **Right Ankle Dorsiflexion (deg) 9** | **Right Ankle Dorsiflexion (deg) 10** | **Right Ankle Dorsiflexion (deg) 11** | **Right Ankle Dorsiflexion (deg) 12** | **Right Ankle Dorsiflexion (deg) 13** | **Right Ankle Dorsiflexion (deg) 14** | **Right Ankle Dorsiflexion (deg) 15** |
| **Exp.** | 4.57 ± 0.51 | 5.48 ± 0.58 | 6.66 ± 1.59 | 8.04 ± 1.78 | 6.90 ± 1.30 | 7.24 ± 0.83 | 7.79 ± 1.46 | 8.09 ± 1.24 | 7.46 ± 1.11 | 6.73 ± 0.86 | 5.84 ± 0.68 | 5.68 ± 0.59 | 5.55 ± 0.65 | 6.13 ± 0.55 | 6.35 ± 0.92 |
| **Control** | 7.61 ± 2.59 | 7.48 ± 2.31 | 8.37 ± 2.61 | 8.45 ± 3.01 | 8.25 ± 2.08 | 7.74 ± 1.65 | 8.31 ± 2.27 | 6.68 ± 1.41 | 8.50 ± 2.50 | 8.20 ± 2.14 | 8.68 ± 2.54 | 8.76 ± 2.47 | 7.69 ± 1.99 | 7.70 ± 1.84 | 8.62 ± 2.45 |
| | **Peak Concentric Force (N) 1** | **Peak Concentric Force (N) 2** | **Peak Concentric Force (N) 3** | **Peak Concentric Force (N) 4** | **Peak Concentric Force (N) 5** | **Peak Concentric Force (N) 6** | **Peak Concentric Force (N) 7** | **Peak Concentric Force (N) 8** | **Peak Concentric Force (N) 9** | **Peak Concentric Force (N) 10** | **Peak Concentric Force (N) 11** | **Peak Concentric Force (N) 12** | **Peak Concentric Force (N) 13** | **Peak Concentric Force (N) 14** | **Peak Concentric Force (N) 15** |
| **Exp.** | 1608.77 ± 99.05 | 1596.75 ± 105.55 | 1596.36 ± 103.80 | 1572.48 ± 101.05 | 1589.44 ± 103.84 | 1601.89 ± 97.02 | 1585.79 ± 96.60 | 1631.32 ± 98.58 | 1579.25 ± 99.77 | 1608.12 ± 100.23 | 1621.24 ± 95.54 | 1585.30 ± 97.95 | 1601.04 ± 91.81 | 1611.39 ± 101.39 | 1576.04 ± 99.21 |
| **Control** | 1595.63 ± 93.86 | 1582.69 ± 99.41 | 1588.13 ± 100.19 | 158335 ± 103.61 | 1557.91 ± 98.33 | 1569.46 ± 101.78 | 1579.73 ± 101.98 | 1564.20 ± 95.00 | 1615.93 ± 96.45 | 1556.74 ± 96.48 | 1547.70 ± 94.52 | 1514.51 ± 85.57 | 1536.95 ± 90.99 | 1499.85 ± 90.56 | 1501.49 ± 93.21 |
| | **Peak Landing Force (N) 1** | **Peak Landing Force (N) 2** | **Peak Landing Force (N) 3** | **Peak Landing Force (N) 4** | **Peak Landing Force (N) 5** | **Peak Landing Force (N) 6** | **Peak Landing Force (N) 7** | **Peak Landing Force (N) 8** | **Peak Landing Force (N) 9** | **Peak Landing Force (N) 10** | **Peak Landing Force (N) 11** | **Peak Landing Force (N) 12** | **Peak Landing Force (N) 13** | **Peak Landing Force (N) 14** | **Peak Landing Force (N) 15** |
| **Exp.** | 2296.21 ± 190.71 | 2107.97 ± 173.62 | 2281.98 ± 199.00 | 2214.86 ± 175.95 | 2264.76 ± 177.77 | 2191.89 ± 210.85 | 2267.70 ± 190.96 | 2312.52 ± 172.07 | 2137.87 ± 149.76 | 2256.31 ± 183.03 | 2155.90 ± 178.36 | 2309.51 ± 208.92 | 2216.08 ± 168.09 | 2199.55 ± 189.36 | 2305.96 ± 148.83 |
| **Control** | 2349.18 ± 186.15 | 2468.16 ± 241.13 | 2425.73 ± 196.38 | 2413.61 ± 189.49 | 2312.59 ± 155.30 | 2382.81 ± 182.19 | 2416.36 ± 176.45 | 2362.83 ± 146.57 | 2305.29 ± 160.34 | 2369.26 ± 173.99 | 2391.49 ± 188.92 | 2271.03 ± 187.71 | 2269.65 ± 166.90 | 2255.65 ± 178.82 | 2258.35 ± 164.93 |

Exp = Experimental; deg = degree.
